# Case Report: Crohn’s disease with coexisting mesenteric schwannoma

**DOI:** 10.3389/fmed.2025.1621976

**Published:** 2025-07-29

**Authors:** Juntao Lu, Yuqi Qiao, Tianrong Wang, Zhijun Cao

**Affiliations:** Division of Gastroenterology and Hepatology, Key Laboratory of Gastroenterology and Hepatology, Ministry of Health, State Key Laboratory for Oncogenes and Related Genes, Renji Hospital, School of Medicine, Shanghai Jiao Tong University, Shanghai Institute of Digestive Disease, Shanghai, China

**Keywords:** Crohn’s disease, mesenteric schwannoma, multidisciplinary team, advanced imaging, surgical section

## Abstract

This case report discusses a rare instance of Crohn’s disease coexisting with a mesenteric schwannoma in a 22-year-old man. The patient initially presented with painful defecation and recurrent fever. Diagnostic procedures, including MRI, CTE, and FDG-PET/CT, revealed signs consistent with Crohn’s disease and an enlarged abdominal “lymph node” suspected to be a schwannoma. Despite exclusive enteral nutrition, the nodule persisted, complicating further treatment. Surgical resection confirmed the diagnosis of schwannoma. This report highlights the diagnostic challenges and treatment complexities when Crohn’s disease coexists with rare tumors such as schwannomas.

## Introduction

Crohn’s disease is a chronic inflammatory bowel condition marked by transmural inflammation and granulomas. Schwannomas, on the other hand, are benign peripheral nerve sheath tumors that are exceedingly rare in the mesenteric region. The coexistence of Crohn’s disease and mesenteric schwannoma presents unique diagnostic and therapeutic challenges. This case report aims to detail the clinical journey, diagnostic hurdles, and treatment considerations in managing a patient with this rare dual pathology.

## Case description

**Figure 1:**
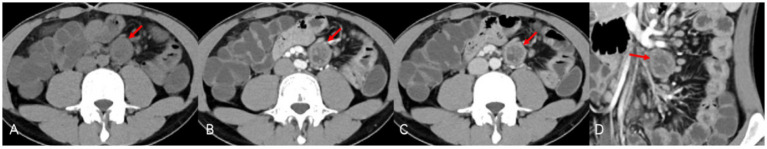
CTE demonstrated inflammation of the small bowel typical of Crohn’s disease. However, precontrast CT **(A)** also revealed an oval-shaped nodule (red arrows) of equal or low density at the root of the left mesentery with clear boundaries, which resembled a significantly enlarged “lymph node.” In postcontrast CT [**(B)** portal venous phase, **(C)** venous phase, and **(D)** portal venous phase/coronal], the nodule showed slow progressive enhancement, especially noticeable at the edges, with a cystic area in the center showing no obvious enhancement.

A 22-year-old male university student has presented with painful defecation and recurrent fever for half a year. Preoperative examination for anal fissure and hemorrhoids revealed an ulcer in the ileocecal region. He was first admitted on 30 April 2024. Symptoms included bowel movements every 2 days, sometimes unformed, with occasional blood in the stool, but no abdominal pain. MRI indicated suspected inflammation in the perianal area, and CTE showed thickened bowel walls and enlarged mesenteric lymph nodes, suggesting inflammatory bowel disease (IBD). Colonoscopy revealed ulcers and edematous mucosa, with a high probability of Crohn’s disease. Biopsy confirmed active chronic ileitis, granulomas, and chronic colitis with mild activity. Due to a significantly enlarged “lymph node” (3 cm) in the abdominal cavity (Figure 1), further evaluation was needed despite a negative T-SPOT test. Exclusive enteral nutrition was administered.

### Timeline of the care

The timeline of the care is summarized in Table 1, which will be further elaborated in the next part.

**Table 1:** Timeline of the care for the case.

Times of admission	Time	Diagnostic assessment	Findings	Interventions recommended
First	May 2024	CTE, colonoscopy, and FDG-PET/CT	Confirmed Crohn’s disease with a significantly enlarged “lymph node” (3 cm) in the abdominal cavity	Entire enteral nutrition for 2 months before re-evaluation
Second	July 2024	CTE, Enhanced MRI	Confirmed Crohn’s disease with a schwannoma or an enlarged necrotic lymph node?	Surgical resection
		Surgical resection	Confirmed Crohn’s disease with a schwannoma (resected)	Commencement of ustekinumab for Crohn’s disease

### Diagnostic assessment, therapeutic intervention, follow-up, and outcomes

FDG-PET/CT on 20 May 2024, indicated significant thickening and increased FDG metabolism in the terminal ileum and other intestinal areas, consistent with IBD, as well as significant lymph node enlargement with increased FDG metabolism. Additional findings included tonsillitis, localized pleural thickening, and reactive bone marrow hyperplasia. Multidisciplinary team (MDT) discussion recommended against immediate surgical intervention due to the risks and suggested continued nutrition therapy for 2 months with re-evaluation.

**Figure 2:**
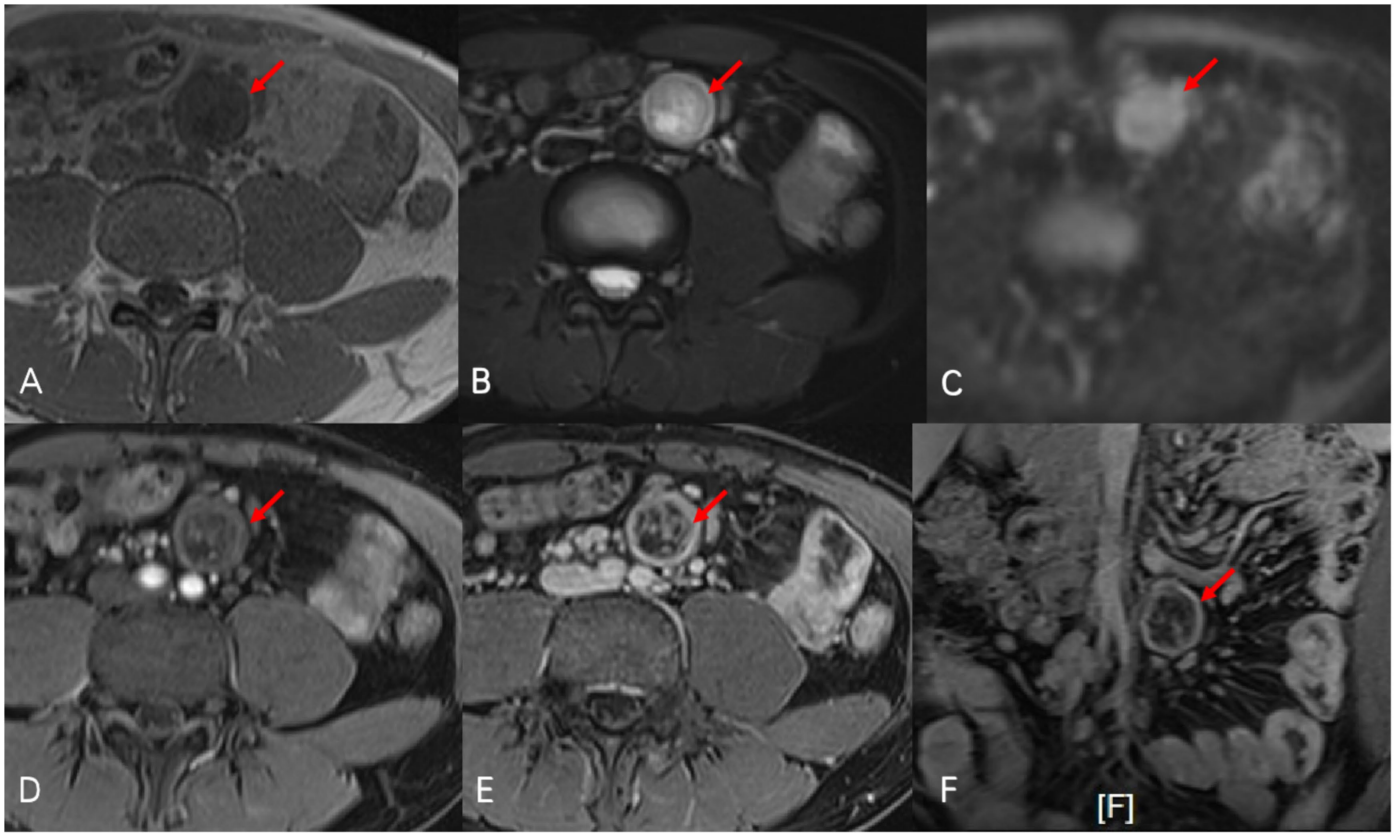
Oval-shaped abnormal signal nodule (red arrow) was observed at the mid-left root of the mesentery in abdominal enhanced MRI. On T1-weighted images **(A)**, the nodule presented as isointense to hypointense, and on fat-suppressed T2-weighted images **(B)**, it showed slightly to markedly higher signals with mixed intensity at the boundaries. The signal is mildly increased on diffusion-weighted imaging **(C)**. After enhancement [**(D)** arterial phase, **(E)** portal venous phase, and **(F)** portal venous phase/coronal], the nodule showed progressive enhancement, with pronounced enhancement at the edges, and a clear cystic change could be seen in the central area.

**Figure 3:**
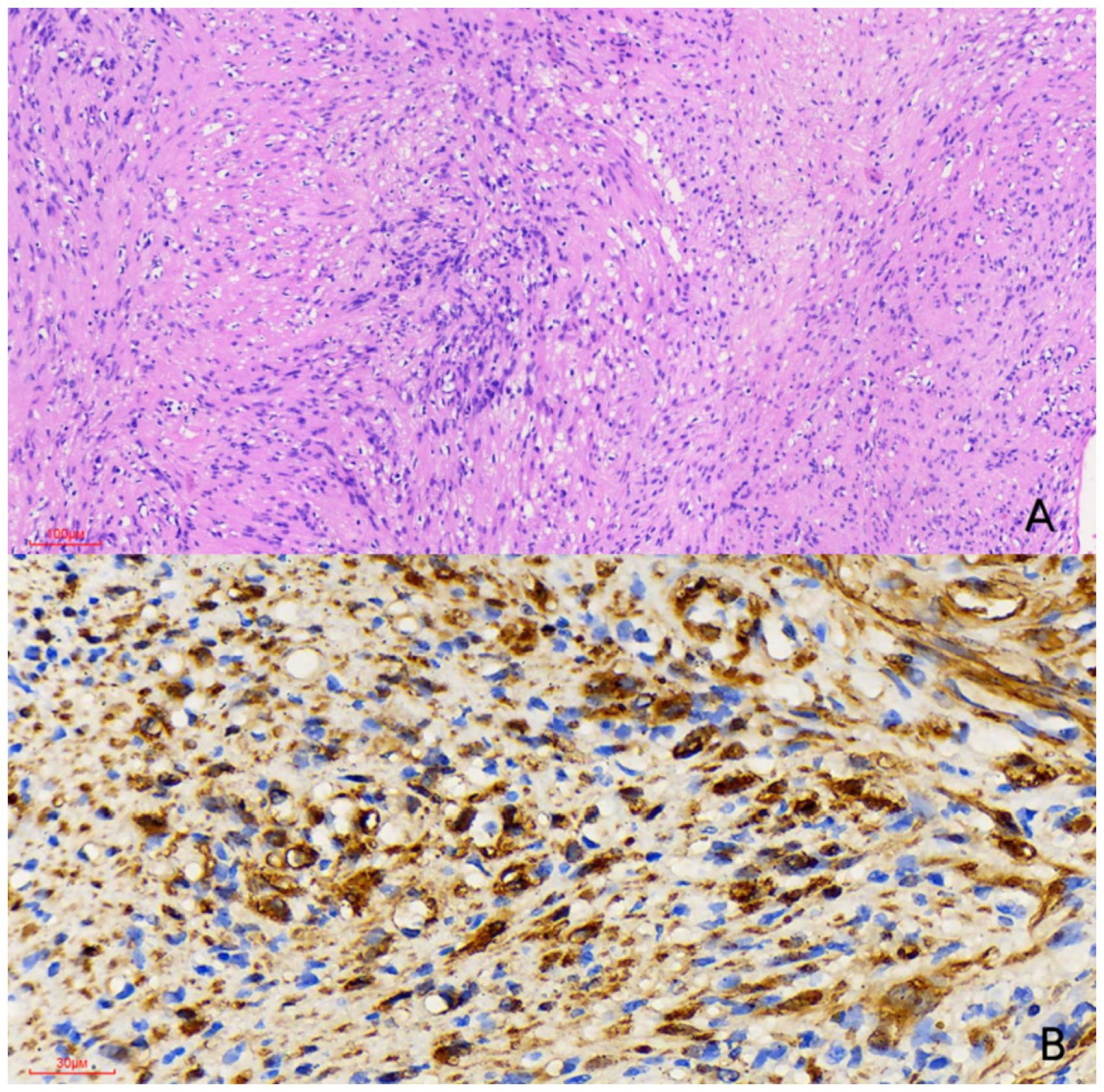
Histological examination [**(A)** hematoxylin and eosin stain, × 100] reveals a spindle cell tumor featuring a biphasic pattern with hypercellular and hypocellular myxoid areas (Antoni A and Antoni B regions). Immunohistochemical examination [**(B)** × 400] showed that the tumor cells were strongly positive for S-100 protein. Therefore, the nodule was diagnosed as a schwannoma.

Two months later, the patient’s symptoms significantly improved with no further fever. CTE on 22 July 2024, showed unchanged thickening and coarsening of the bowel walls, multiple full mesenteric lymph nodes, and a persistent cystic-solid nodule near the left common iliac artery, potentially a schwannoma. Enhanced MRI on 25 July 2024, confirmed these findings, with the nodule resembling an enlarged necrotic lymph node (Figure 2). Due to the unclear pathological diagnosis, tuberculosis, lymphoma, and other diseases could not be excluded, complicating the treatment of Crohn’s disease with biologics or immunosuppressants. MDT discussion on 29 July 2024 recommended surgical resection over percutaneous biopsy due to potential risks and complications. Surgery revealed a hard, smooth nodule (3 × 3 cm) in the mesentery above the left iliac artery. Preliminary pathology suggested a spindle cell tumor, and immunohistochemistry on 8 August 2024, confirmed it as a schwannoma with the following features: CD117 (−), CD34 (−), DOG-1 (−), SMA (−), S-100 (+), Desmin (−), Ki67 (+ 1%), SDHB (+), EMA (−), and BRAF (−) (Figure 3). The patient recovered well after surgery and was initiated on ustekinumab therapy for Crohn’s disease. Follow-up evaluations at 6 months and 1 year revealed no signs of schwannoma recurrence, with slight improvement in Crohn’s disease compared to the pre-treatment status.

## Discussion

Schwannomas are benign peripheral nerve sheath tumors originating from Schwann cells, with mesenteric schwannomas being extremely uncommon. Clinically, they often present nonspecifically or asymptomatically, discovered incidentally during imaging or routine checks (1, 2). When symptomatic, patients may experience abdominal pain, a palpable mass, gastrointestinal obstruction, or other nonspecific symptoms such as nausea, vomiting, and weight loss (2–6).

The coexistence of Crohn’s disease and schwannoma is exceedingly rare, with only two reported cases and none involving mesenteric schwannoma (7, 8). This coexistence poses unique diagnostic and therapeutic challenges due to the differing histological features of Crohn’s disease and schwannomas. Previous literature suggests a potential interaction between inflammatory and tumor processes. Pellino et al. hypothesized that TGF-*β* might influence the pathogenesis of both diseases by affecting S100A4 expression and fibroblast migration (8). Schwannomas could overlap with radiological findings of Crohn’s disease, such as multiple enlarged lymph nodes, complicating differential diagnoses with conditions such as lymphoma, special infections, and tuberculosis.

Diagnosing mesenteric schwannomas is challenging due to their rarity and non-specific clinical and radiological features. Imaging modalities such as ultrasound (US), CT, MRI, and FDG-PET/CT are crucial.

The US is suitable for preliminary screening and monitoring, observing the tumor’s size, shape, and position (2, 3, 9–11). Schwannomas typically appear as hypoechoic areas on US, aiding differentiation from other tumors. The US can guide fine-needle aspiration biopsy for pathological examination (12). However, the US is limited by imaging depth and operator skills, and it may not be able to detect deeper or asymptomatic tumors.

CT is a primary diagnostic tool for both Crohn’s disease and mesenteric schwannomas, which often present as a well-defined, hypodense mass (2, 5, 9–15). However, CT characteristics of schwannomas are nonspecific and may resemble those of other soft tissue tumors. Bae et al. found regional lymph node enlargement in 71.7% of schwannoma cases, complicating the diagnosis (1). Our patient’s CT scans suggested a schwannoma but could not rule out lymph node enlargement, which has different clinical significance in Crohn’s disease.

MRI is also essential for diagnosing mesenteric schwannomas, with low signal intensity on T1-weighted images and high signal intensity on T2-weighted images (2, 5, 10–12). Gadolinium-enhanced MRI reveals the tumor’s vascularity, aiding differentiation from other tumor (9, 13). Despite the characteristic MRI features, other tumors may exhibit similar signals, making preoperative diagnosis challenging. In our case, MRI suggested the possibility of enlarged necrotic lymph nodes.

FDG-PET/CT provides information about tumor metabolic activity, helping distinguish schwannomas from other tumors such as GISTs and carcinomas (12, 16). However, it is not definitive, as other tumors may show similar FDG accumulation. In our patient, the differential diagnosis also included tuberculosis and lymphoma, justifying the use of FDG-PET/CT.

MDT discussions are pivotal in managing complex Crohn’s disease cases, especially those with unexplained abdominal masses. Differential diagnosis must consider special infections, such as tuberculosis and malignant tumors, such as lymphoma. We consulted radiology, nuclear medicine, surgery, interventional radiology, pathology, and infectious diseases departments. The consensus was that histopathological examination is the gold standard for diagnosis. While the interventional procedure may help clarify the pathology, it cannot achieve complete resection. Surgical intervention, despite its risks, offers the possibility of complete mass removal. In our case, surgical resection confirmed the mass as a schwannoma and achieved therapeutic goals.

Uncertainty remains in treating Crohn’s disease when concurrent with schwannomas. No guidelines or case reports address this scenario, making the use of biologics and immunosuppressants particularly challenging. Although schwannomas are benign, there is a risk of recurrence (15). Enteral nutrition is a safe and effective treatment for Crohn’s disease in such cases, but the long-term safety of biologics and immunosuppressants is unknown. Close monitoring and individualized treatment are crucial to managing these uncertainties.

### Patient perspective

The patient, well-informed during the whole process, was encouraged to participate in every step of decision-making. Now he has started ustekinumab for treatment of Crohn’s disease. He appraised the transparency of our medical care as well as the efficient and comprehensive communication between the patient and medical staff.

## Conclusion

The coexistence of Crohn’s disease and mesenteric schwannomas is rare, requiring a multidisciplinary approach for diagnosis and treatment. Advanced imaging (CT, MRI, and FDG-PET/CT) is key for identification, with pathology confirming the diagnosis. Surgical resection remains the definitive treatment. Further studies and case reports are needed to better understand and manage these complex cases.

## Data Availability

The original contributions presented in the study are included in the article/supplementary material, further inquiries can be directed to the corresponding author.
